# Supplementary material for: Comprehensive Annotation and Functional Exploration of MicroRNAs in Lettuce
Source: Front Plant Sci. 2021 Dec 24;12:781836. doi: 10.3389/fpls.2021.781836 (PMC8739914; doi:10.3389/fpls.2021.781836)
Supplement: Supplementary file 3 [file Data_Sheet_9.ZIP › GSM2230752.plot/Lsa-miR396c_Lsat_1_v5_gn_5_72841.1_2120_TPlot.pdf]

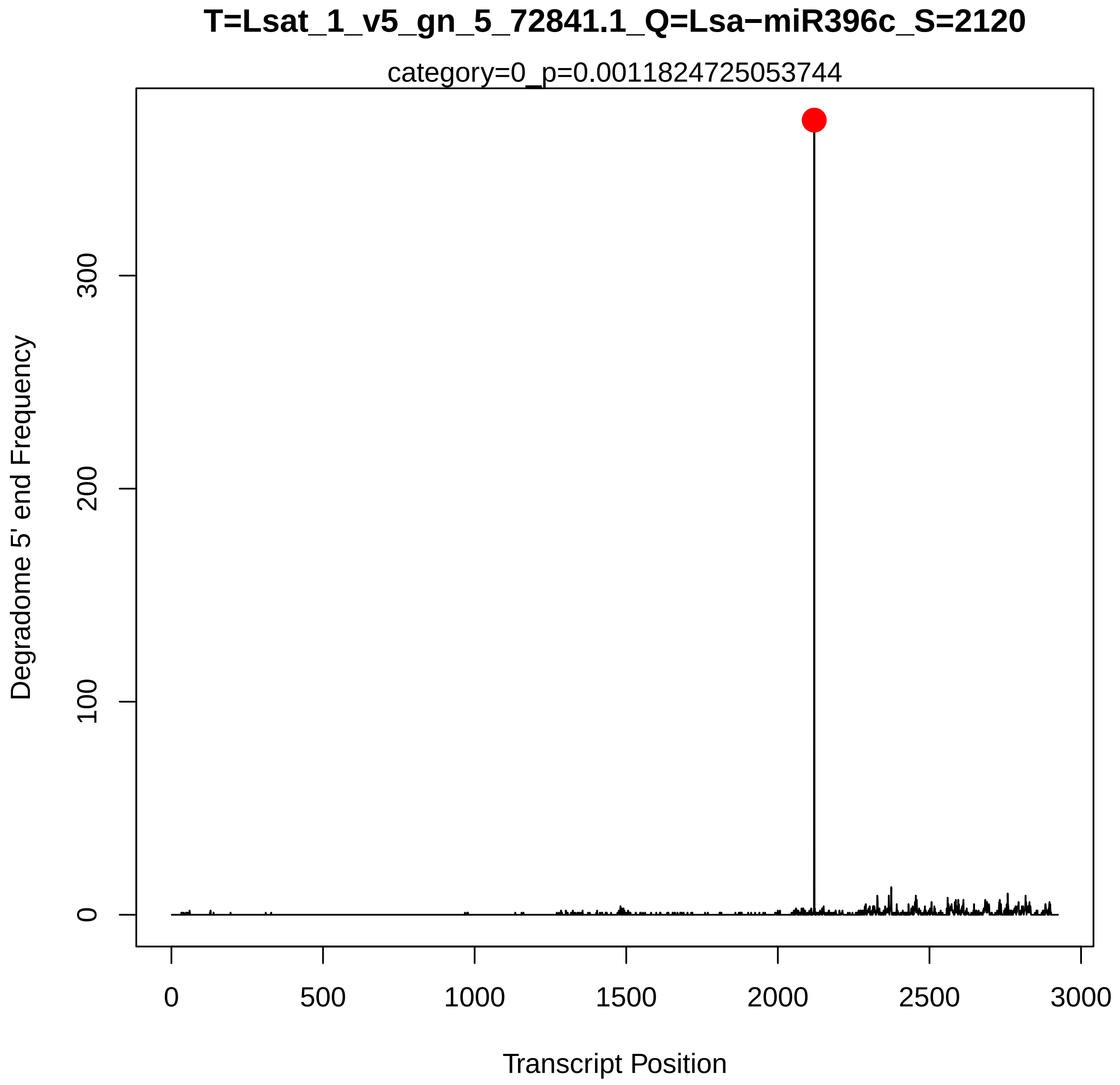

T=Lsat_1_v5_gn_5_72841.1_Q=Lsa−miR396c_S=2120
category=0_p=0.00118247250537744
Degradome 5' end Frequency
0
100
200
300
0
500
1000
1500
2000
2500
3000
Transcript Position
